# Fluoroquinolones in Drug-Resistant Tuberculosis: Culture Conversion and Pharmacokinetic/Pharmacodynamic Target Attainment To Guide Dose Selection

**DOI:** 10.1128/AAC.00279-19

**Published:** 2019-06-24

**Authors:** Mohammad H. Al-Shaer, Wael A. Alghamdi, Abdullah Alsultan, Guohua An, Shahriar Ahmed, Yosra Alkabab, Sayera Banu, Ketevan Barbakadze, Eric Houpt, Maia Kipiani, Lali Mikiashvili, J. Peter Cegielski, Russell R. Kempker, Scott K. Heysell, Charles A. Peloquin

**Affiliations:** a Infectious Disease Pharmacokinetics Laboratory, College of Pharmacy and Emerging Pathogens Institute, University of Florida, Gainesville, Florida, USA; b Department of Clinical Pharmacy, College of Pharmacy, King Khalid University, Abha, Saudi Arabia; c Department of Clinical Pharmacy, College of Pharmacy, King Saud University, Riyadh, Saudi Arabia; d Division of Pharmaceutics and Translational Therapeutics, College of Pharmacy, University of Iowa, Iowa City, Iowa, USA; e Infectious Diseases Division, International Centre for Diarrhoeal Diseases Research (ICDDR,B), Dhaka, Bangladesh; f Division of Infectious Diseases and International Health, Department of Medicine, University of Virginia, Charlottesville, Virginia, USA; g National Center for TB and Lung Diseases, Tbilisi, Georgia; h University of Texas Health Science Center at Tyler, Tyler, Texas, USA; i Division of Infectious Diseases, Department of Medicine, Emory University, Atlanta, Georgia, USA

**Keywords:** Monte Carlo simulation, fluoroquinolones, multidrug resistance, pharmacodynamics, population pharmacokinetics, tuberculosis

## Abstract

Fluoroquinolones are group A drugs in tuberculosis guidelines. We aim to compare the culture conversion between new-generation (levofloxacin and moxifloxacin) and old-generation (ciprofloxacin and ofloxacin) fluoroquinolones, develop pharmacokinetic models, and calculate target attainment for levofloxacin and moxifloxacin. We included three U.S. tuberculosis centers. Patients admitted between 1984 and 2015, infected with drug-resistant tuberculosis, and who had received fluoroquinolones for ≥28 days were included. Demographics, sputum cultures and susceptibility, treatment regimens, and serum concentrations were collected. A time-to-event analysis was conducted, and Cox proportional hazards model was used to compare the time to culture conversion. Using additional data from ongoing studies, pharmacokinetic modelling and Monte Carlo simulations were performed to assess target attainment for different doses. Overall, 124 patients received fluoroquinolones. The median age was 40 years, and the median weight was 60 kg. Fifty-six patients (45%) received old-generation fluoroquinolones. New-generation fluoroquinolones showed a faster time to culture conversion (median 16 versus 40 weeks, *P* = 0.012). After adjusting for isoniazid and clofazimine treatment, patients treated with new-generation fluoroquinolones were more likely to have culture conversion (adjusted hazards ratio, 2.16 [95% confidence interval, 1.28 to 3.64]). We included 178 patients in the pharmacokinetic models. Levofloxacin and moxifloxacin were best described by a one-compartment model with first-order absorption and elimination. At least 1,500 to 1,750 mg levofloxacin and 800 mg moxifloxacin may be needed for maximum kill at the current epidemiologic cutoff values. In summary, new-generation fluoroquinolones showed faster time to culture conversion compared to the old generation. For optimal target attainment at the current MIC values, higher doses of levofloxacin and moxifloxacin may be needed.

## Abstract (precis)

Fluoroquinolones are group A drugs in tuberculosis guidelines. We aim to compare the culture conversion between new-generation (levofloxacin and moxifloxacin) and old-generation (ciprofloxacin and ofloxacin) fluoroquinolones, develop pharmacokinetic models, and calculate target attainment for levofloxacin and moxifloxacin. We included three U.S. tuberculosis centers.

## INTRODUCTION

Tuberculosis (TB) has impacted human health for many millennia (1). Currently, active TB disease affects >10 million people and kills over 1.7 million people annually, making it the most lethal infectious agent worldwide. These trends have been improving slightly for the last few years (2). However, when data are stratified based on resistance, isoniazid- and rifampin-resistant, or multidrug-resistant TB (MDR-TB), cases are increasing, and most cases are not reported (3). In addition, the outcomes of treating MDR-TB are much worse compared to drug-susceptible TB, as demonstrated by the global treatment success rates of 55 and 85%, respectively (2).

There are currently numerous TB drugs under development and different combinations are being tested in clinical trials. Fluoroquinolones (FQs) are considered an essential part of an MDR-TB regimen. Recently, the World Health Organization (WHO) has changed the priority of certain drugs in the treatment of MDR-TB, but FQs remained in group A (4). Initially, ciprofloxacin (CIP) and ofloxacin (OFL) were used for TB treatment because they were approved earlier, in 1987 and 1992, respectively; then, later-generation FQs, levofloxacin (LVX) and moxifloxacin (MOX), were found to be associated with higher treatment success and lower mortality in patients who received them compared to those who did not (5). The later-generation FQs were compared in many studies, in which both MOX and LVX were shown to have good penetration into cavitary lesions (6, 7). The animal and *in silico* efficacy data favored MOX, but clinical data showed that both FQs have similar outcomes (8–13). Differences in these observations may be driven by pharmacokinetic variability in humans that was not adequately represented in the preclinical studies. Furthermore, limited data are available on the comparison of newer- versus older-generation FQs that incorporate pharmacokinetic/pharmacodynamic (PK/PD) assessment. As such, current dosing regimens for the later generation of FQs may need to be optimized in order to achieve PK/PD targets associated with optimal *in vitro* microbial kill and clinical treatment outcome in MDR-TB patients (14–17).

Therefore, we compared time to culture conversion between the old (CIP and OFL) and new (LVX and MOX) generations of FQs based on retrospective data obtained from three U.S. centers where serum drug concentrations were measured commonly for clinical care. We also developed population PK models for MOX and LVX utilizing rich PK sampling from ongoing studies among MDR-TB patients from geographically diverse settings and performed target attainment analysis with the goal of dose optimization.

## RESULTS

### Culture conversion cohort.

A total of 124 MDR-TB patients from the U.S. hospitals in the retrospective cohort received fluoroquinolones. Eleven patients were reported to have FQ-resistant TB, and two of them remained culture positive until the end of follow-up. The median age (range) was 40 years (15 to 93), the median weight was 60 kg (37 to 105), and the majority were males (69%). Fifty-six patients (45%) received the older-generation of FQs, CIP or OFL (Table 1).

**TABLE 1:** Patient demographics from the three U.S. centers (AGH, TCID, and UTHSCT)

Characteristic^*a*^	Median (range) or % (no.) (*n* = 124)
Age, yr	39.5 (15.0–93.0)
Sex, male	69.4 (86)
Wt, kg	59.9 (37.0–105.0)
FQ received	
OFL/CIP	45.2 (56)
LVX/MOX	54.8 (68)
Cavitary disease	17.7 (22)
Extrapulmonary TB	8.1 (10)
HIV	12.9 (16)
Diabetes	22.6 (28)
Lung disease^*b*^	24.2 (30)

a FQ, fluoroquinolone; LVX/MOX, levofloxacin/moxifloxacin; OFL/CIP, ofloxacin/ciprofloxacin; TB, tuberculosis.

b Including chronic obstructive pulmonary disease, asthma, bronchiectasis, silicosis, asbestosis, and pneumonectomy.

**FIG 1:**
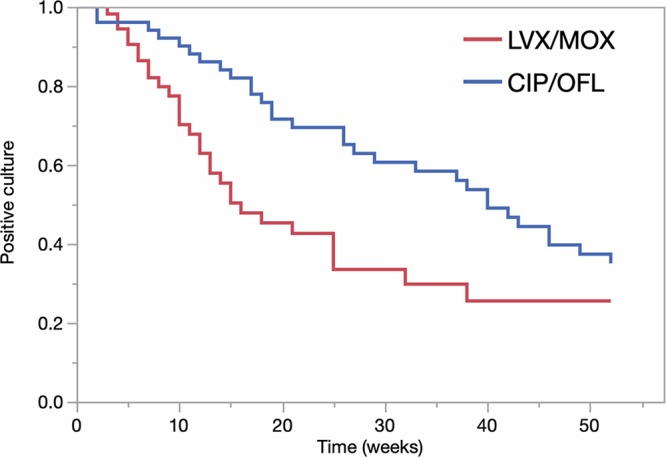
Kaplan-Meier curve comparing time to culture conversion between LVX/MOX and CIP/OFL in MDR-TB patients. CIP/OFL, ciprofloxacin/ofloxacin; LVX/MOX, levofloxacin/moxifloxacin.

After excluding patients with initial negative cultures, 106 patients were included in the time-to-event (TTE) analysis (Fig. 1). LVX/MOX showed faster time to culture conversion in MDR-TB patients compared to CIP/OFL (median, 16 versus 40 weeks; log-rank *P = *0.012). After excluding FQ-resistant TB patients, the median time to culture conversion was 12 (LVX/MOX) versus 36 (CIP/OFL) weeks (*P < *0.0001). Using Cox proportional hazards regression model, the bivariate analysis revealed seven potential covariates for inclusion in the final model: lung disease, cancer, aminoglycoside resistance, and concurrent use of isoniazid, clofazimine, and linezolid (Table 2). However, only isoniazid (*P = *0.0041) and clofazimine (*P = *0.0048) were significant once included in the final model. As a result, the final model included the FQ treatment group, isoniazid, and clofazimine, and showed that the culture conversion was faster with LVX/MOX group (adjusted hazards ratio [aHR], 2.16 [95% confidence interval {CI}, 1.28 to 3.64]). For the other two covariates in the final model, culture conversion was slower with concurrent isoniazid use (aHR, 0.35 [95% CI, 0.16 to 0.78]) and faster with clofazimine concurrent use (aHR, 2.51 [95% CI, 1.37 to 4.60]). Among patients receiving isoniazid (*n* = 29), only eight patients received high-dose isoniazid, which was not a significant covariate in the model.

**TABLE 2:** Preliminary and final Cox proportional hazards models^*a*^

Factor	Preliminary model		Final model	
	Hazard ratio	95% CI	Adjusted hazard ratio	95% CI
New-generation FQs	**1.91**	**1.14–3.21**	**2.16**	**1.28–3.64**
Lung disease^*b*^	**0.51**	**0.28–0.96**		
Cancer	1.60	0.81–3.15		
AMG resistance	1.38	0.80–2.39		
Concurrent anti-TB drugs				
Isoniazid	**0.34**	**0.16–0.75**	**0.35**	**0.16–0.78**
Clofazimine	**2.97**	**1.63–5.43**	**2.51**	**1.37–4.60**
Linezolid	1.77	0.89–3.51		

a AMG, aminoglycoside; CI, confidence interval; FQs, fluoroquinolones; TB, tuberculosis. Values in boldface indicate *P* values of <0.05.

b Including chronic obstructive pulmonary disease, asthma, and bronchiectasis.

### Population PK models and simulations.

For the population PK models, 30 and 36 patients from the U.S. centers had drug concentrations and were included in the LVX and MOX models, respectively. The other sites contributed a total of 78 patients to LVX and 34 patients to MOX. The total number of samples included in the LVX and MOX PK models were 553 and 312, respectively. Plasma concentrations across the sampling intervals ranged from 0.3 to 43.0 mg/liter for LVX and from 0 to 12.8 mg/liter for MOX. Table 3 shows the combined demographics for all patients included in the models.

**TABLE 3:** Combined demographics of all patients included in the models (AGH, TCID, Brazil, Georgia, and Bangladesh)^*a*^

Characteristic	Mean (SD) or % (no.)	
	Levofloxacin (*n* = 108)	Moxifloxacin (*n* = 70)
Age, yr	41.8 (14.2)	40.1 (15.4)
Sex, male	77.8 (84)	81.4 (57)
Wt, kg	58.1 (14.3)	57.5 (13.6)
Dose, mg/kg	14.7 (3.3)	10.4 (6.2)
SrCr, mg/dl	0.91 (0.29)	0.92 (0.55)
CL_CR_, ml/min	89.7 (30.9)	93.6 (40.5)

a CL_CR_, creatinine clearance; SrCr, serum creatinine.

**FIG 2:**
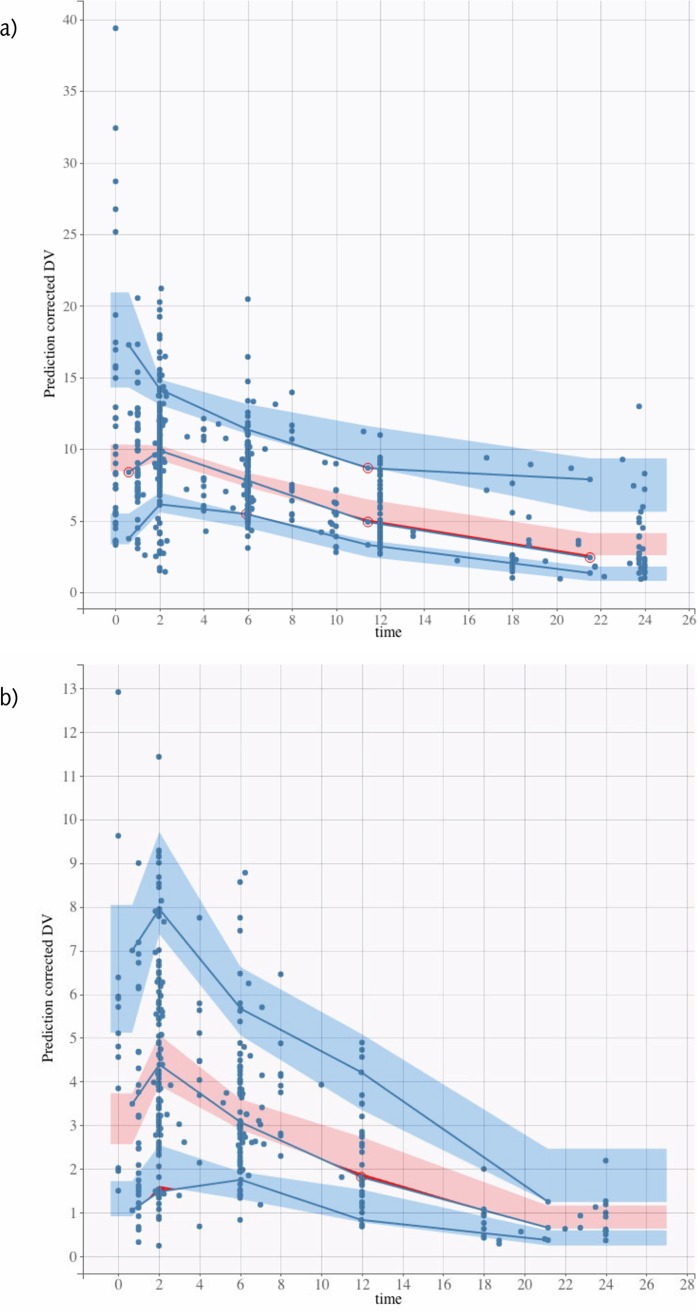
Visual predictive checks for levofloxacin (a) and moxifloxacin (b).

Models for both LVX and MOX were best described by a one-compartment model with first-order absorption and elimination. Proportional residual error model was used. For LVX, creatinine clearance (CL_CR_) had a significant effect on apparent clearance (CL/F), and sex and weight had a significant effect on apparent volume of distribution (*V*/F). All were included in the model. The effect of body weight on *V*/F was fixed to 1 in the final model. For MOX, no covariates influenced the PK parameters significantly. However, *V*/F and CL/F random effects were correlated. Figures S1 and S2 in the supplemental material show the observations versus individual and population predictions, and Fig. 2 shows the visual predictive checks for each model. The parameter estimates of the final models are presented in Table 4.

**TABLE 4:** Parameter estimates for the final models

Parameter^*a*^	Levofloxacin			Moxifloxacin		
	Estimate	SE	RSE (%)	Estimate	SE	RSE (%)
Fixed effects						
*K_a_* (h^−1^)	2.95	0.80	27.00	2.69	1.54	57.20
*V*/F (liters)	60.50	3.09	5.10	110	9.10	8.25
CL/F (liters/h)	6.22	0.22	3.54	9.59	0.47	4.87
Beta, sex (M) on *V*/F^*b*^	0.36	0.05	15.00			
Beta, wt on *V*/F	1	—	—			
Beta, CL_CR_ on CL/F^*c*^	0.52	0.10	19.50			
						
Random effects						
Omega, ka	1.40	0.20	14.50	1.96	0.49	25.20
Omega, *V*/F	0.13	0.03	24.60	0.38	0.07	18.20
Omega, CL/F	0.18	0.07	39.40	0.30	0.04	12.50
Gamma, CL/F	0.29	0.04	15.00			
						
Correlations						
*V*/F and CL/F				0.79	0.12	15.30
						
Error model parameters						
Proportional	0.19	0.01	4.15	0.34	0.02	5.06

a *K_a_*, absorption rate constant; Beta, estimated effect; CL/F, apparent clearance; CL_CR_, creatinine clearance; gamma, inter-occasion variability; omega, interindividual variability; *V*/F, apparent volume of distribution.

b *P* = 2.93 × 10^−11^.

c *P* = 3.12 × 10^−7^.

**FIG 3:**
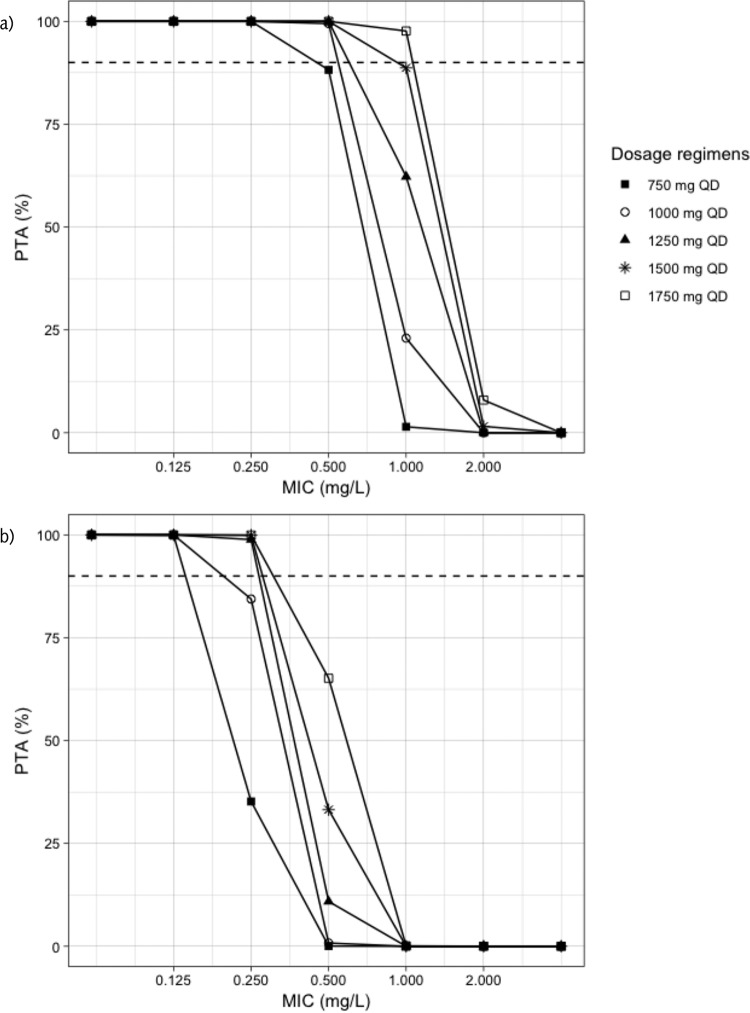
Probability of target attainment for levofloxacin. PTA, probability of target attainment; QD, once daily. (a) Target is *f*AUC_0–24_/MIC 130; (b) target is *f*AUC_0–24_/MIC 360. The dashed line corresponds to 90% target attainment.

**FIG 4:**
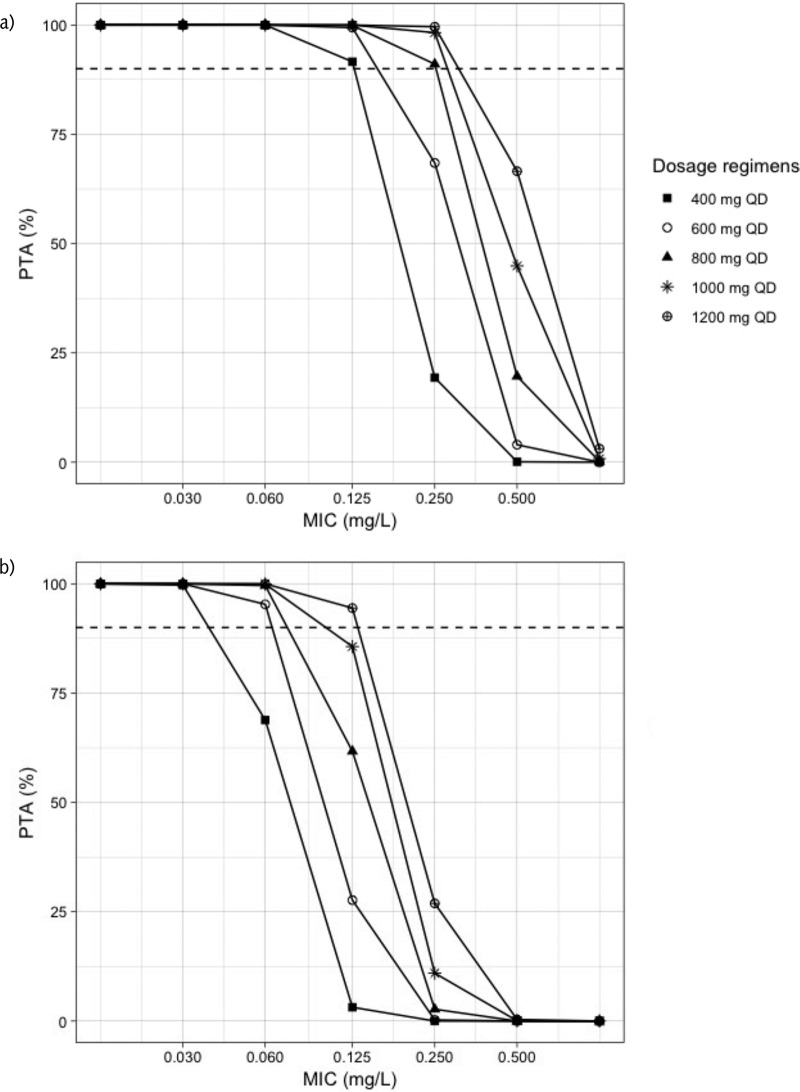
Probability of target attainment for moxifloxacin. PTA, probability of target attainment; QD, once daily. (a) Target is *f*AUC_0–24_/MIC 130; (b) target is *f*AUC_0–24_/MIC 360. The dashed line corresponds to 90% target attainment.

Figure 3 shows the probability of target attainment (PTA) for LVX. For the target free area under the concentration-time curve from 0 to 24 h to MIC ratio (*f*AUC_0–24_/MIC) of 130 associated with maximal kill, all the regimens achieved more than 90% PTA at MIC of 0.25 mg/liter. At an MIC of 0.5 mg/liter, PTA was more than 90% for all doses, with the exception of the 750-mg regimen, which had PTA of 88%. Only 1,750 mg achieved PTA higher than 90% at MIC of 1 mg/liter, while 1,500 mg achieved a target of 89%. For a *f*AUC_0–24_/MIC of 360 associated with the suppression of resistance, all the regimens achieved PTA higher than 90% at an MIC of 0.125 mg/liter, and none of the regimens achieved 90% PTA for resistance suppression when the MIC was 0.5 mg/liter or higher. The results of target attainment analysis for MOX are shown in Fig. 4. For the *f*AUC_0–24_/MIC 130, all the simulated dosing regimens achieved at least 90% PTA at MIC of 0.125 mg/liter, while at least 800 mg was needed to achieve the same target at an MIC of 0.25 mg/liter. None of the regimens achieved a PTA of 90% at an MIC of 0.5 mg/liter or higher. Similarly, the 90% PTA for the resistance suppression at *f*AUC_0–24_/MIC 360 was achieved by 600 mg or higher for an MIC of 0.06 mg/liter, while for an MIC of 0.125 mg/liter, only 1,200 mg daily achieved the target. None of the regimens achieved the resistance suppression target at an MIC of 0.25 mg/liter or more. Table 5 summarizes the simulated doses and the PK/PD breakpoints.

**TABLE 5:** Simulated exposure and PK/PD breakpoints^*a*^

Simulated dose (mg/day)	Mean *f*AUC_0–24_ (SD), mg ⋅ h/liter	PK/PD breakpoint (mg/liter)^*b*^	
		*f*AUC_0–24_/MIC 130	*f*AUC_0–24_/MIC 360
Levofloxacin			
750	84.98 (17.88)	0.25	0.125
1,000	113.61 (23.65)	0.50	0.125
1,250	142.76 (29.41)	0.50	0.25
1,500	168.97 (35.33)	0.50	0.25
1,750	199.27 (41.56)	1.00	0.25
			
Moxifloxacin			
400	26.23 (8.37)	0.125	0.03
600	39.32 (12.57)	0.125	0.06
800	52.15 (16.72)	0.25	0.06
1,000	65.06 (20.17)	0.25	0.06
1,200	77.72 (25.11)	0.25	0.125

a PK/PD, pharmacokinetic/pharmacodynamic. *f*AUC_0–24_, area under the free concentration-time curve from time zero to 24 h.

b The PK/PD breakpoint is the highest MIC when at least 90% target attainment is achieved.

## DISCUSSION

We performed culture conversion analysis based on data from MDR-TB patients from U.S. TB centers that allowed comparison of regimens containing older- and newer-generation FQs, while combining these data with studies from other geographically diverse countries for population PK modeling of LVX and MOX to inform optimal dose strategies. Unsurprisingly, we found regimens containing LVX or MOX showed a faster time to culture conversion compared to those containing CIP or OFL. However, the magnitude of the difference was profound. There may be several reasons for our observations. Seifert et al. used Cox proportional hazards model to compare the mortality between 834 MDR-TB patients, who received later-generation FQs (MOX or LVX), and those who received no or earlier-generation FQs (OFL or sparfloxacin). The model was adjusted for human immunodeficiency virus (HIV) status, study site, body mass index, and phenotypic resistance profile, which showed that use of MOX/LVX was associated with lower risk of mortality compared to the other group (aHR, 0.46 [95% CI, 0.26 to 0.80]) (18). However, these researchers did not control for concurrent TB drugs. In our study, we used the time to culture conversion as the outcome instead of mortality and excluded patients not receiving FQs, and the result favored the use of the later-generation FQs. These findings are important given that the time to culture conversion in MDR-TB patients at 6 months of therapy were found significantly associated with treatment success compared to failure or death (19). We also found that concurrent isoniazid and clofazimine use significantly influenced our model. Importantly, clofazimine was associated with a shorter time to culture conversion, which is consistent with the recent prioritization included in WHO revision of MDR-TB treatment (4). Another retrospective study was conducted on 40 and 59 MDR-TB patients who received LVX and OFL, respectively. The time to culture conversion and the incidence of adverse events were similar among groups; however, success rate was higher with the LVX group (odds ratio, 4; *P = *0.049) (20).

Different *in vitro* pharmacodynamic targets have been suggested for the FQs in TB, including *f*AUC_0–24_/MIC values of >101, 132, and 146 for maximal kill (21–23). These targets achieved under different experimental conditions are similar, and we performed our analysis using a target of 130 given that it is the closest to the one tested under acidic pH in mycobacteria at an MIC of 0.5 mg/liter (21). Recently, Deshpande et al. reported a much higher resistance suppression target of *f*AUC_0-24_/MIC >360 (22) and, based on our simulation, was unreachable by even the highest suggested doses at the current epidemiologic cutoff (ECOFF) values on conventional media.

Using a model-based approach and Monte Carlo simulation, Zvada et al. showed that MOX achieved better target attainment and cumulative fraction of response, especially when the simulated dose was 800 mg, compared to OFL at 800 mg (16). In our study, only 12 patients received MOX at 800 mg, which may suggest that it was not a usual practice to give such dose at that time. Nevertheless, the MOX/LVX group was still showing higher efficacy, which presented as a faster time to culture conversion compared to CIP/OFL. Our target attainment analysis showed that a 750- to 1,000-mg dose or higher of LVX is needed to achieve at least 90% of PTA associated with maximum kill at MIC of 0.5 mg/liter, while all regimens at the same MIC failed to achieve the resistance suppression target. In addition, at the ECOFF value of 1 mg/liter, only 1,750 mg achieved the maximum kill target, while 1,500 mg approached the target, which may suggest that higher doses of LVX are needed for an MIC of 1 mg/liter. Similarly, MOX of at least 800 mg daily was needed to achieve 90% of PTA associated with maximum kill at an MIC of 0.25 mg/liter, while all regimens failed to achieve the resistance suppression target at the same MIC. This has been emphasized previously, and it was shown that patients who received LVX achieved a *f*AUC/MIC ≥100 only when the MICs were 0.25 and 0.5 mg/liter, and doses greater than 15 mg/kg would be needed for better target attainment (14). In a review on optimization of LVX in MDR-TB patients, 80% of patients who received 1,000 mg per day with an MIC of 1 mg/liter did not achieve a *f*AUC/MIC >100 (15). Similarly, “Opti-Q” was a double-blinded, randomized, dose-ranging clinical trial conducted on 101 patients with MDR-TB. The patients received LVX at 11, 14, 17, or 20 mg/kg/day and achieved median AUC_0–24_ values of 109, 98, 145, and 207 mg ⋅ h/liter, respectively, suggesting that dose increase produces a relatively linear exposure increase (24). Recently, the STREAM trial has reported a higher number of patients developing QTc prolongation in the short, high-dose MOX regimen compared to the long, conventional one (31 [11%] versus 9 [6%], *P* = 0.14) (25). Ongoing prospective cohorts designed with intensive PK/PD assessment among MDR-TB patients on combination drug regimens will likely provide further insight into translating the preclinical PTA values for microbial kill and resistance suppression into clinical targets. Importantly, the vast majority of settings where MDR-TB is endemic do not have access to MIC testing. While *gyrA* mutation can explain the majority of strains with phenotypic resistance to LVX and MOX, it is those strains wild-type to *gyrA* by conventional line-probe assays or with such low levels of mutant populations as to only be detected by next-generation sequencing that may have “susceptible” MICs near the ECOFF, but for whom PK variability renders them well below the 90% PTA (26, 27). Ultimately, therapeutic drug monitoring is needed in these patients to optimize therapy.

Our study has a number of limitations. There were no PK data for the earlier-generation FQs group, which prevented including these data in the TTE analysis and Cox hazard model, although the accumulating evidence favors the use of later-generation over the old-generation FQs (5). In addition, only the total concentrations were reported and, hence, we had to apply a fixed unbound fraction to all the concentrations. The AUC/MIC optimal target is not yet well defined. We followed a conservative approach by selecting a high target for maximal kill and resistance suppression. Also, sampling bias might be present since some of the U.S. centers used to request drug concentrations only for difficult-to-treat MDR-TB cases. Finally, we did not look at the safety of the high simulated FQ doses whose tolerability is questionable.

In conclusion, in MDR-TB patients, LVX and MOX showed faster time to culture conversion compared to CIP and OFL. LVX and MOX were well described using one-compartment models while including CL_CR_, sex, and weight as covariates in the LVX model. Current guidelines do not address FQ dose based on PK/PD evidence, but our data support renewed attention to quantitative susceptibility testing (28). Higher doses of LVX and MOX may be needed for maximum kill at the ECOFF values of 1 and 0.25 mg/liter, respectively, and such dosing prioritizes the need for access to individualized therapeutic drug monitoring in MDR-TB endemic settings.

## MATERIALS AND METHODS

### Culture conversion cohort.

This was a multicenter, retrospective study which included data from three TB centers in the United States: A. G. Holley Hospital (AGH), Texas Centre for Infectious Diseases (TCID), and University of Texas Health Science Centre at Tyler (UTHSCT). We included patients admitted between 1984 and 2015, infected with pulmonary rifampin-resistant or MDR-TB, and who received an FQ for at least 4 weeks. Patients demographics, sputum cultures, susceptibility data, duration of treatment, and FQ random serum concentrations were collected. A TTE analysis was conducted to compare the time to culture conversion among FQs (CIP/OFL versus LVX/MOX). The time was defined as the number of weeks from the start of treatment to culture conversion. Culture conversion was defined as two consecutive negative cultures with no positive culture thereafter. Patients were censored if their last culture was positive and/or the time to culture conversion was more than 52 weeks (1 year). Patients who had negative cultures from the start were excluded from the TTE analysis.

Continuous data were presented as means (standard deviations [SD]) or medians (ranges) and categorical data as counts and percentages. Kaplan-Meier curves and the log-rank test were used to compare time to culture conversion between FQs groups. Cox proportional hazards models were used to determine the aHRs for the TTE analysis. Initially, bivariate analyses were performed for the following covariates: sex, age, body mass index, cavitary disease, extrapulmonary disease, HIV, diabetes, cancer, lung disease, liver disease, aminoglycoside/FQ resistance, and TB treatment received for at least 28 days. All covariates with a *P* value of <0.25 in the bivariate analysis were included in the preliminary multivariable model. The final model included only covariates with *P* value of <0.05. Statistical tests were performed using JMP Pro v14.0 (SAS Institute, Cary, NC).

### Population PK modeling and simulations cohort.

Population PK models for LVX and MOX were established using PK data from patients who had at least one drug concentration in the present study from the AGH and TCID sites. The dose ranges were 250 to 1,250 mg of LVX and 400 to 800 mg of MOX. The average sampling times were 3 h for LVX and 4 h for MOX. We also included PK data from other studies conducted in Brazil (29), Georgia, and Bangladesh (NCT03559582). The study conducted in Brazil was a randomized trial in TB patients who received 1,000 mg LVX or 400 mg MOX and had blood samples drawn before and 1, 2, 4, 8, 12, 18, and 24 h after the fifth dose (29). In Georgia, a prospective study was completed in which patients received 750 to 1,000 mg LVX or 400 mg MOX and had PK samples collected before and 2, 6 to 8, 10 to 12, and 24 h after receiving the dose 4 to 6 weeks after initiating treatment. Finally, patients in the prospective study in Bangladesh received LVX at 500 to 1,000 mg or MOX at 400 to 800 mg, and samples were collected at 1, 2, 6, and 12 h after receiving the dose during week 2 of therapy and at 2 and 6 h during weeks 4 and 6 of therapy.

The plasma samples were stored at –80°C until the time of quantification. The drug quantification in the plasma samples collected in the prospective studies was done at the Infectious Disease Pharmacokinetics Laboratory (University of Florida) using a validated liquid chromatography tandem mass spectrometry assay. The analysis was performed on Thermo Scientific TSQ Endura or TSQ Quantum Ultra. The curve was linear over the range from 0.2 to 15 mg/liter. Samples with concentrations above the range were diluted and reanalyzed. The interbatch precision was 0.38 to 2.98% for LVX and 1.02 to 3.30% for MOX. The intrabatch and interbatch accuracy ranges were 94.10 to 104.63% and 96.20 to 103.62% for LVX and 106.28 to 114.52% and 108.79 to 113.90% for MOX, respectively. A validated assay was used to quantify drugs in samples from Brazil as described by Peloquin et al. (29). For the retrospective data from the U.S. centers, drug concentrations were collected from the patient medical records.

The PK model was built in a stepwise manner, including developing the structure model, adding the stochastic model to describe the variability within populations using multiple levels of random effects, and finally testing the significance of potential covariates. Several structural models were tested, including one- and two-compartment models, and first-order absorption and elimination were evaluated. Interindividual variability in parameters was estimated using an exponential model. Residual variability was evaluated using the additive, proportional, or combination of additive and proportional error models. The covariates investigated for influence on PK parameters included age, sex, body weight, and CL_CR_. Covariate analysis was performed using the standard forward addition and backward elimination method. Forward addition was applied first to determine significant covariates. Only covariates that decreased the −2 log-likelihood (–2LL) by more than 3.84 compared to the base model were considered for the full covariate selection. Backward elimination was then applied to remove covariates from the model with an increase in the –2LL of >6.63.

Using the final parameter estimates from the models, we performed Monte Carlo simulation (MCS) for a total of 10,000 patients for each drug. For LVX, we simulated 750, 1,000, 1,250, 1,500, and 1,750 mg once-daily dosing regimens. We used an MIC range of 0.125 to 2.00 mg/liter (14, 30). For MOX, 400-, 600-, 800-, 1,000-, and 1,200-mg once-daily regimens were simulated, and an MIC range of 0.03 to 0.50 mg/liter was used (31). We calculated the *f*AUC_0–24_, assuming an unbound drug fraction of 70% for LVX and 60% for MOX (31). For the PTA calculation, we used PK/PD targets of ≥130 and ≥360 for the *f*AUC_0–24_/MIC, representing the maximum kill and suppression of resistance, respectively (21, 22). PK modeling was done using Monolix v2018R1 (Antony, France: Lixoft SAS, 2018), and MCS was performed using mlxR package v3.3.0 in R software v3.5.1.

The included studies were approved by the Institutional Review Boards (IRBs) at the participating sites (AGH IRB 2014-12, Emory University IRB 00083639, ICDDR,B: IRB PR-15121, NCTLD: IRB 00007705, TCID: IRB 14-013, University of Florida IRB: 201300638, University of Virginia: IRB 18452, UTHSCT IRB 09-016). For the prospective studies, written informed consents were obtained from all participants or their legal guardians. For the retrospective studies, informed consent was waived.

## Supplementary Material

Supplemental file 1
