# Supplementary material for: Fluoroquinolones in Drug-Resistant Tuberculosis: Culture Conversion and Pharmacokinetic/Pharmacodynamic Target Attainment To Guide Dose Selection
Source: Antimicrob Agents Chemother. 2019 Jun 24;63(7):e00279-19. doi: 10.1128/AAC.00279-19 (PMC6591615; doi:10.1128/AAC.00279-19)
Supplement: Supplemental file 1 [file AAC.00279-19-s0001.pdf]

Supplemental Material

Figure S1: Levofloxacin observations vs. (a) individual (b) population predictions

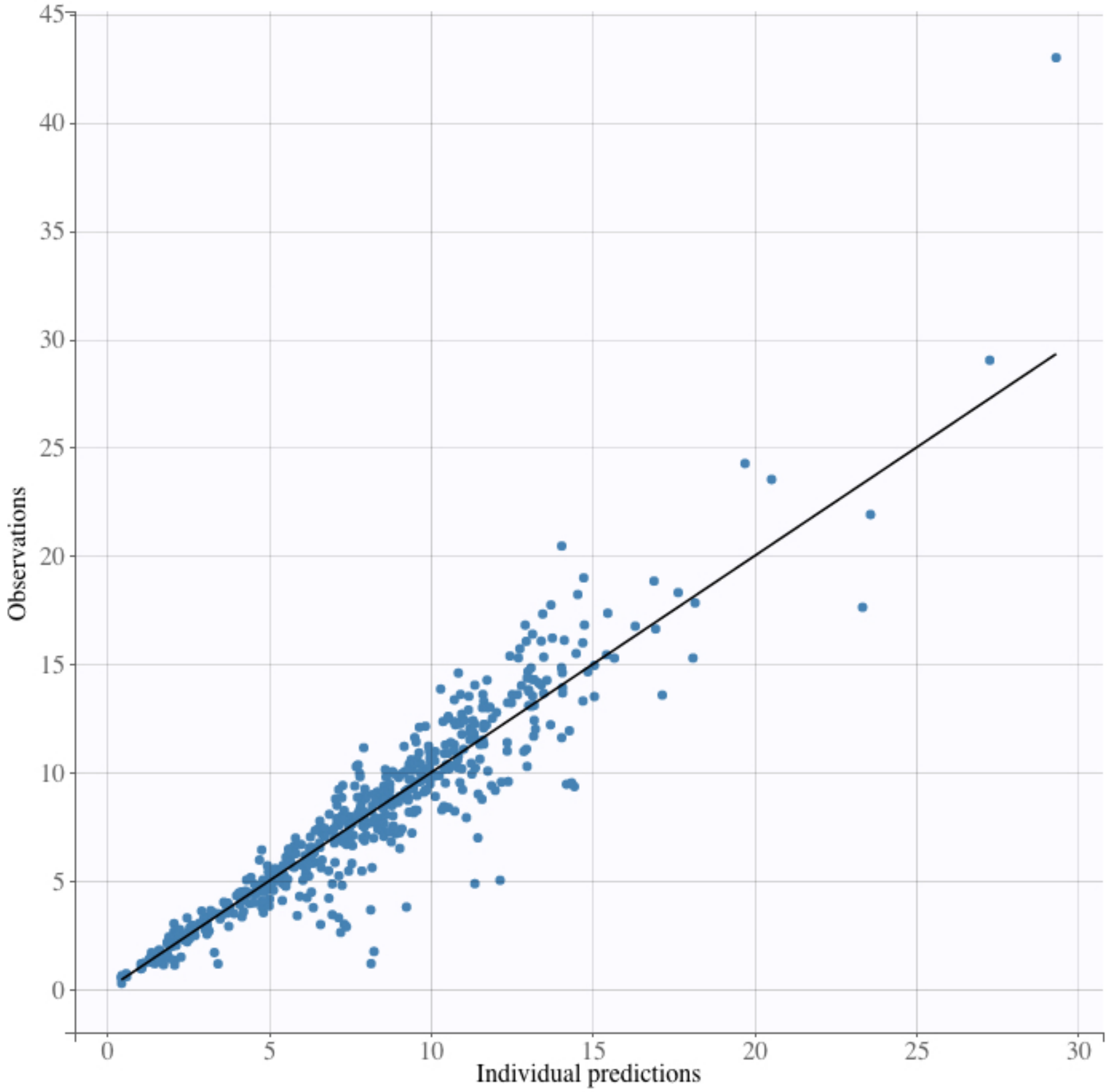

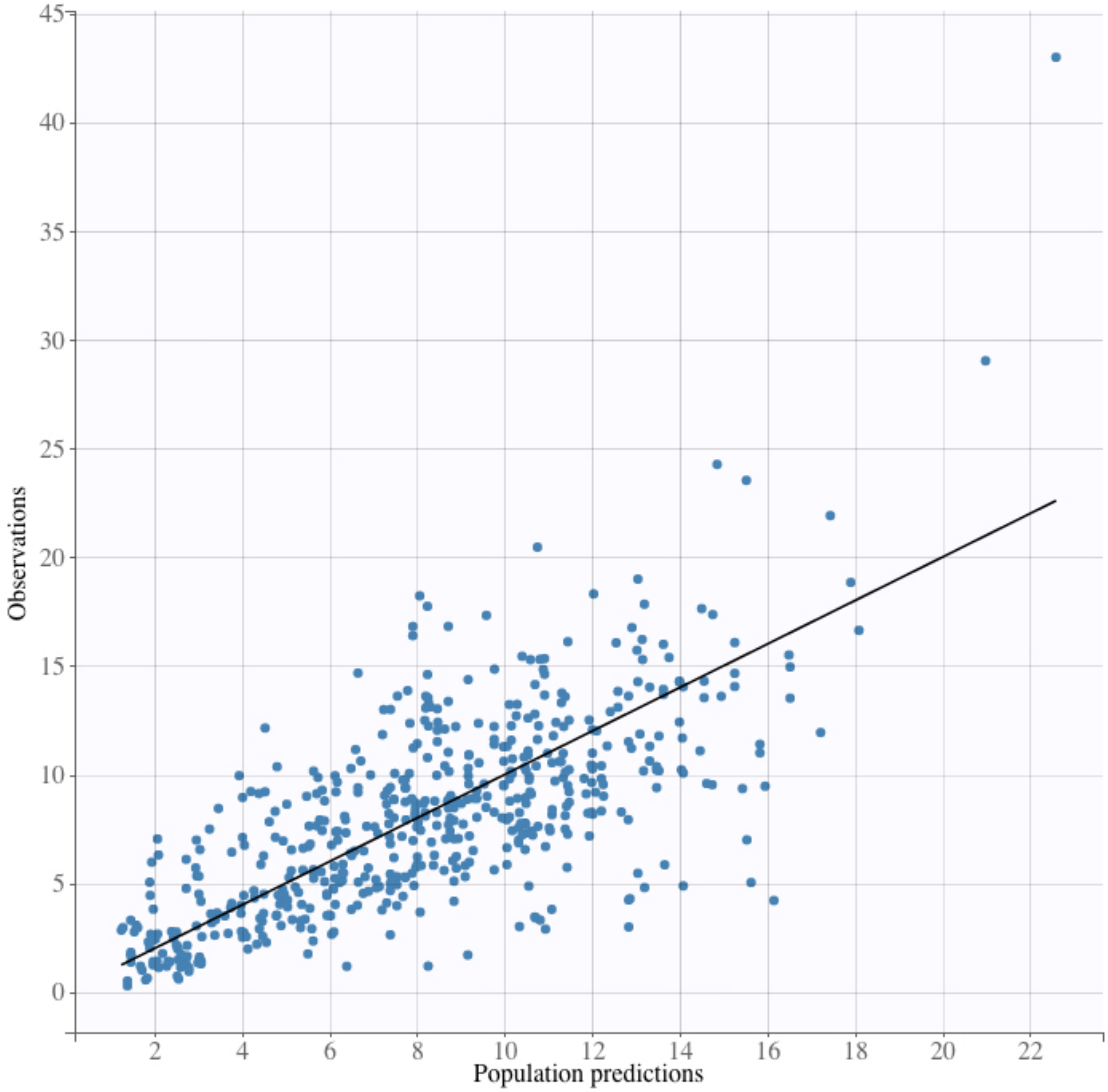
Observations
Population predictions
0
5
10
15
20
25
30
35
40
45
2
4
6
8
10
12
14
16
18
20
22

Figure S2: Moxifloxacin observations vs. (a) individual (b) population predictions

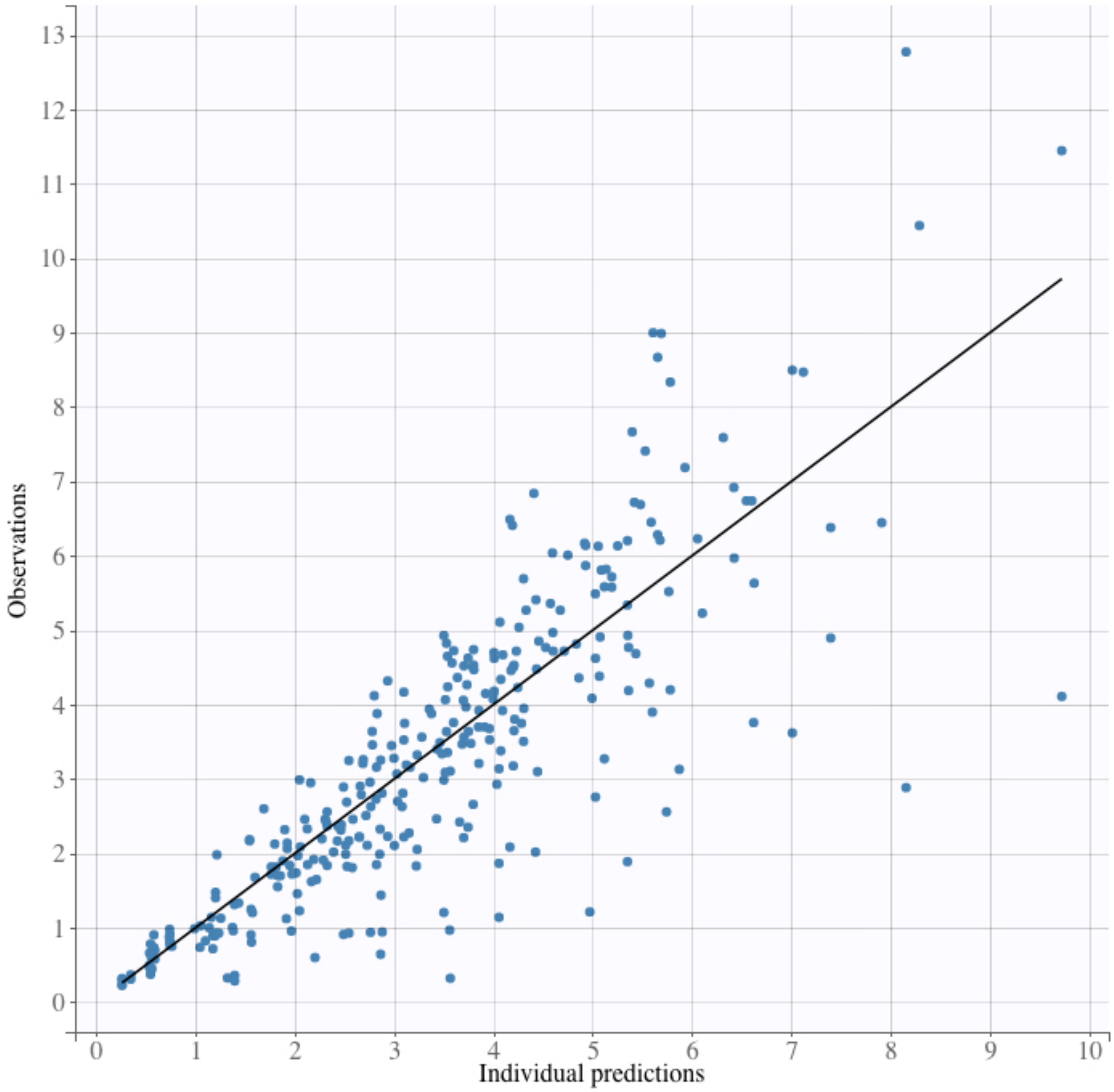

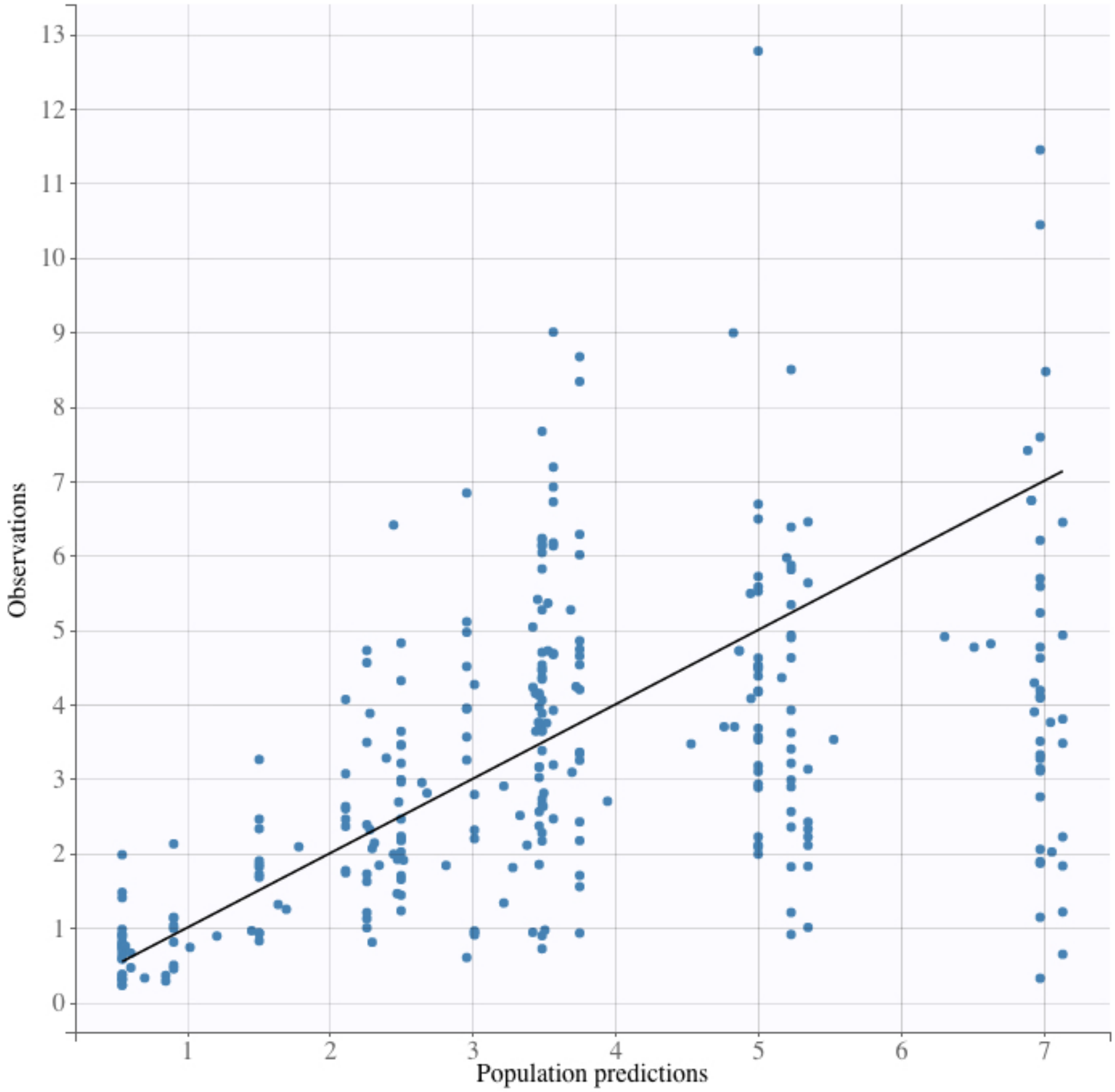
Observations
Population predictions
0
1
2
3
4
5
6
7
8
9
10
11
12
13
